# Genotypic Distribution and Phylogenetic Characterization of *Enterocytozoon bieneusi* in Diarrheic Chickens and Pigs in Multiple Cities, China: Potential Zoonotic Transmission

**DOI:** 10.1371/journal.pone.0108279

**Published:** 2014-09-25

**Authors:** Wei Li, Wei Tao, Yanxue Jiang, Ruinan Diao, Jinping Yang, Lihua Xiao

**Affiliations:** 1 College of Veterinary Medicine, Northeast Agricultural University, Harbin, Heilongjiang, China; 2 Division of Foodborne, Waterborne and Environmental Diseases, Centers for Disease Control and Prevention, Atlanta, Georgia, United States of America; Technion-Israel Institute of Technology Haifa 32000 Israel, Israel

## Abstract

This study investigated diarrheic broiler and layer chickens (<50 days; n = 14) and pigs of three age groups (preweaned <30 days, weaned ≈30 to 60 days, and growing >60 days; n = 64) for *E. bieneusi* genotypes in northeast China and evaluated the potential roles of chickens and pigs in zoonotic transmission of microsporidiosis. Two 45-day-old layer chickens in city Jixi, Heilongjiang province and one 23-day-old broiler chicken in city Songyuan, Jilin province were identified to harbor a human-pathogenic *E. bieneusi* genotype Henan-IV and a new genotype named CC-1, respectively, by nested PCR and sequence analysis of the ribosomal internal transcribed spacer (ITS). Eleven of 64 (17.2%) duodenal mucosal specimens from pigs in city Tianjin, city Tongliao of Inner Mongolia, cities Jilin and Songyuan of Jilin province, and cities Daqing, Harbin, and Suihua of Heilongjiang province, were positive for *E. bieneusi*, with the infection rates of weaned pigs (35%, 7/20) significantly higher than preweaned ones (3.6%, 1/28; P<0.05). Nucleotide sequences of the ITS were obtained from 6 pig specimens, belonging to 3 known genotypes CHN7, EbpC, and Henan-IV. That the previous reports have described the occurrence of genotypes EbpC and Henan-IV in humans and EbpC in wastewater in central China and the clustering of genotypes CC-1 and CHN7 into a major phylogenetic group of *E. bieneusi* genotypes with zoonotic potential indicated that chickens and pigs could be potential sources of human micorsporidiosis. To our knowledge, this is the first report describing the existence of zoonotic *E. bieneusi* genotypes in diarrheic chickens.

## Introduction

Microsporidia, spore-forming unicellular parasites, are comprised of approximately 150 genera and over 1200 species and recently reclassified as fungi, of which *Enterocytozoon bieneusi* is the most common species infecting humans. *E. bieneusi* is also an emerging enteric pathogen leading to diarrhea in a variety of vertebrate animals and even birds [1]–[3]. Humans, wild and domestic animals, and birds have the potential to produce environmentally resistant spores of *E. bieneusi* into water systems and cause public health issues. Unavailability of effective vaccines and medications for *E. bieneusi* highlights the necessity of understanding its epidemiology and subsequently developing preventive measures [1]–[6].

Because of the lack of in vitro culture approaches and the difficulty in morphological distinctness of the spores, a molecular typing tool relying on the polymorphisms of the ribosomal internal transcribed spacer (ITS) is now widely applied for diagnosis and genotyping of *E. bieneusi* [1]–[3]. ITS genotyping has contributed to the identification of over 150 *E. bieneusi* genotypes in broad geographic and host ranges [1], [3]. Phylogenetic analysis using the neighbor-joining method has classified them into five or more genetically isolated groups, with zoonotic genotypes in Group 1 infecting both humans and animals and host-adapted ones specific to animals in several other groups [1], [4], [7].

Despite many advances in the genotypic identification of *E. bieneusi* in humans and wild and domestic animals worldwide, the importance of poultry in zoonotic transmission of microsporidiosis remains unclear. Since the first detection of *E. bieneusi* in chickens in 2002, there have been no infection reports of this organism in poultry [8]. Nevertheless, the detection of human-pathogenic *E. bieneusi* genotypes in pet birds, pigeons, and falcons suggests that birds might play an important role in the transmission of human microsporidiosis [9]–[13]. Thus far, over 40 *E. bieneusi* genotypes have been characterized from swine worldwide, most of which belong to Group 1 and have public health significance [1], [7], [14]–[27].

Although the epidemiology of microsporidiosis in China remains unclear, limited data generated from molecular characteristics of *E. bieneusi* genotypes in domestic animals, humans, and wastewater have been helpful to elucidate the sources and transmission routes of this neglected disease [4], [25], [27]–[29]. This study focused on the identification of *E. bieneusi* genotypes in 14 duodenal mucosal specimens from young farm chickens (<50 days) with acute watery diarrhea in 7 cities in northeast China and 64 duodenal mucosal specimens from severely diarrheic pigs of three age groups (preweaned <30 days, weaned ≈30 to 60 days, and growing >60 days) farmed in city Tianjin and 11 other cities in northeast China, and the evaluation of the potential roles of chickens and pigs in zoonotic transmission of microsporidiosis.

## Materials and Methods

### Ethics statement

This study was performed in accordance with the recommendations in the Guide for the Care and Use of Laboratory Animals of the Ministry of Health, China. Prior to experiment, the protocol of the current study was reviewed and approved by the Institutional Animal Care and Use Committee of Northeast Agricultural University, under the approved protocol number SRM-08. Before beginning work on the study, we contacted the farm owners and obtained their permission. No specific permits were required for the described field studies. And the locations where we sampled are not privately-owned or protected in any way. The field studies did not involve endangered or protected species.

### Clinical specimens

Fourteen duodenal mucosal specimens were obtained from 9 layer chickens (LC) and 5 broiler chickens (BC) (<50 days) with acute watery diarrhea in cities Changchun (1 LC: 20 days), Songyuan (1 BC: 23 days), and Tonghua (1 BC: 13 days) of Jilin Province and Daqing (1 BC: 40 days), Harbin (3 LC: 15, 20, and 27 days; 2 BC: 7 and 31 days), Jixi (4 LC: 5, 10, 45, and 45 days), and Suihua (1 LC: 19 days) of Heilongjiang Province during October 2012 to May 2013.

The specimens of duodenal mucosa was collected from 64 severely diarrheic pigs of three age groups: 28 preweaned pigs (PP) <30 days, 20 weaned pigs (WP) ≈30 to 60 days, and 16 growing pigs (GP) >60 days, in cities Tianjin (3 PP), Chaoyang (1 PP) of Liaoning province, Tongliao (3 PP and 3 WP) of Inner Mongolia, Jilin (1 WP and 2 GP) and Songyuan (2 WP) of Jilin province, and Daqing (2 WP and 1 GP), Harbin (9 PP, 6 WP, and 6 GP), Jixi (3 GP), Jiamusi (7 PP and 1 WP), Qiqihaer (1 PP, 1 WP, and 1 GP), Suihua (3 PP, 4 WP, and 3 GP), and Yichun (1 PP) of Heilongjiang province. The sampling date for 61 pigs were during May 2013 to July 2013, 1 in January 2013, and 2 in April 2013.

One specimen per animal kept free range or housed individually was used in this study. The procedures for preparing and collecting duodenal mucosal specimens were the same as described [25]. The specimens containing duodenal mucosa and contents were collected in 50 ml plastic containers and stored at –20°C for DNA extraction.

### Sample processing

After being washed twice in distilled water, specimens (0.3 g or thereabout) were subjected to DNA extraction using a Stool DNA Rapid Extraction Kit (Spin-column) (BioTeke, China) and manufacturer-recommended procedures. *E. bieneusi*-positive specimens were identified by PCR of a 392-bp product that covered the entire ITS of the rRNA gene using nested primers as described [30]. PCR amplification was performed in an Eppendorf Mastercycler Gradient PCR Thermal Cycler (Eppendorf, Westbury, NY, USA) and PCR results were visualized by electrophoresis in 1.5% agarose containing ethidium bromide.

### Data analysis

The amplicons of anticipated size were sent to the Sangon Company (Shanghai, China) for DNA sequencing in both directions. All raw sequencing data were viewed and proofread in Chromas Pro version 1.33 (Technelysium Pty. Ltd., Helensvale, Queensland, Australia). The resulting DNA sequences were aligned to reference sequences using the ClustalX program package (version 1.81; available from: URL: ftp://ftp-igbmc.u-strasbg.fr/pub/ClustalX/) to determine *E. bieneusi* genotypes. To assess the relationship of *E. bieneusi* genotypes identified herein and those described in previous studies, a neighbor-joining tree rooted with GenBank sequence DQ885585 was constructed using the software Mega 4 (http://www.megasoftware.net/) and the evolutionary distances calculated by Kimura 2-parameter model. Reliability of clustering patterns in the phylogenetic analysis was assessed by the bootstrap method using 1,000 bootstrap replicates.

The infection rates between different age groups was compared by use of a chi-square test at a significance of P<0.05 using software SPSS version 17.0 (SPSS Inc., Chicago, Illinois, USA).

## Results

### Frequency of *E. bieneusi* in chickens and pigs

**Figure 1:**
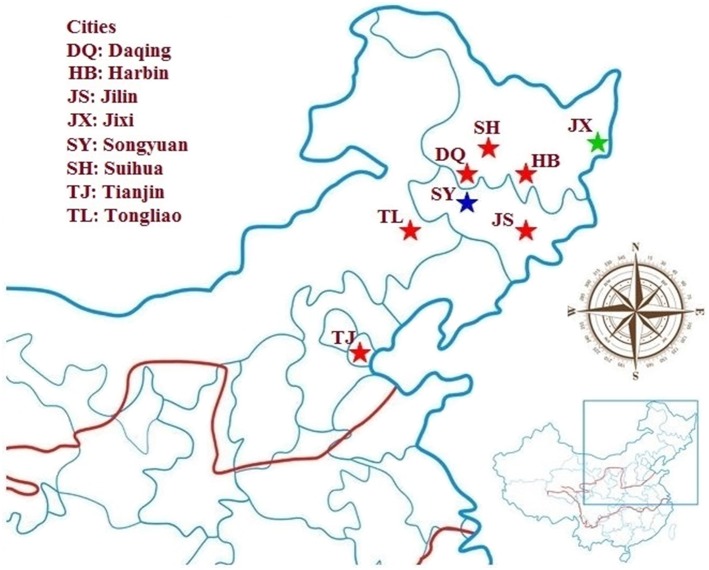
Existence of *Enterocytozoon bieneusi* in multiple cities in China. Red stars: cities (Daqing, Jilin, Harbin, Suihua, Tianjin, and Tongliao) where infections of *E. bieneusi* in pigs were examined; green star: city Jixi where infections of the pathogen in chickens were examined; blue star: city Songyuan where the organism was identified in both chicken and pigs.

Two layer chickens from Jixi (45-day-old) and a broiler chicken from Songyuan (23-day-old) were positive for *E. bieneusi* (Fig. 1).

Two of 2 pigs in Songyuan, 2 of 3 in Jilin, 1 of 3 in Daqing, 1 of 3 in Tianjin, 2 of 10 in Suihua, 1 of 6 in Tongliao, and 2 of 21 in Harbin were identified to be *E. bieneusi*-positive (Fig. 1). We did not detect the pathogen in the other 5 sampled cities. The total infection rate was 17.2% (11/64). Weaned pigs (35%, 7/20) had a significantly higher rate of infection than preweaned pigs (3.6%, 1/28; 0.01<P<0.05, χ^2^ = 6.2), whereas the difference in infection rates between weaned and growing pigs (18.8%, 3/16) or between growing and preweaned pigs was not significant. *E. bieneusi* was detected in 1 of 1 pig sampled in January 2013, 0 of 2 in April 2013, 1 of 19 in May 2013, 3 of 22 in June 2013, and 6 of 20 in July 2013.

### Genotypic distribution

One known *E. bieneusi* genotype Henan-IV was detected in the two layer chickens and a new genotype named CC-1 in a broiler chicken (Table 1). The two distinct genotypes differed from each other by only one single nucleotide in the ITS sequences. Nucleotide sequence of the ITS of the new genotype CC-1 was deposited in the GenBank database under the accession number KF724905.

**Table 1:** *Enterocytozoon bieneusi* genotypes identified in farmed chickens and pigs in China.

Animal	Genotype	Positive no^a^	City (province)^b^	Host (location^c^)	Reference
Chicken	Henan-IV	2 LC	JX (HL)	Human (China) and Pig (China)	[25], [28]
	CC-1	1 BC	SY (JL)		This study
Pig	CHN7	1 WP	TL (IM)	Pig (China)	[27]
	EbpC	2 WP and 1 GP	HB and SH (HL)	Human (China, Vietnam, Thailand,and CZE), Pig (China, Thailand,Japan, Germany, and Switzerland), andWild mammals (China, Austria,CZE, Poland, and USA)	[1], [14], [25], [28], [29], [37], [40]
	Henan-IV	2 WP	SY (JL)	See above	[25], [28]

a LC: layer chickens = 45 days; BC: broiler chickens = 23 days; WP: weaned pigs ≈30 to 60 days; GP: growing pigs >60 days.

b City and province where the genotypes were identified in this study; JX: Jixi; SY: Songyuan; TL: Tongliao; HB: Harbin; SH: Suihua; JL: Jilin; HL: Heilongjiang; IM: Inner Mongolia.

c Location where the genotypes were identified before this work; CZE: Czech Republic.

Nucleotide sequences of the ITS were obtained from 6 of 11 *E. bieneusi*-positive pig specimens, detecting 3 known distinct genotypes CHN7, EbpC, and Henan-IV. Genotype CHN7 was identified in a weaned pig in Tongliao, EbpC in a growing pig in Suihua and 2 weaned pigs in Harbin, and Henan-IV in 2 weaned pigs in Songyuan (Table 1).

### Phylogeny

**Figure 2:**
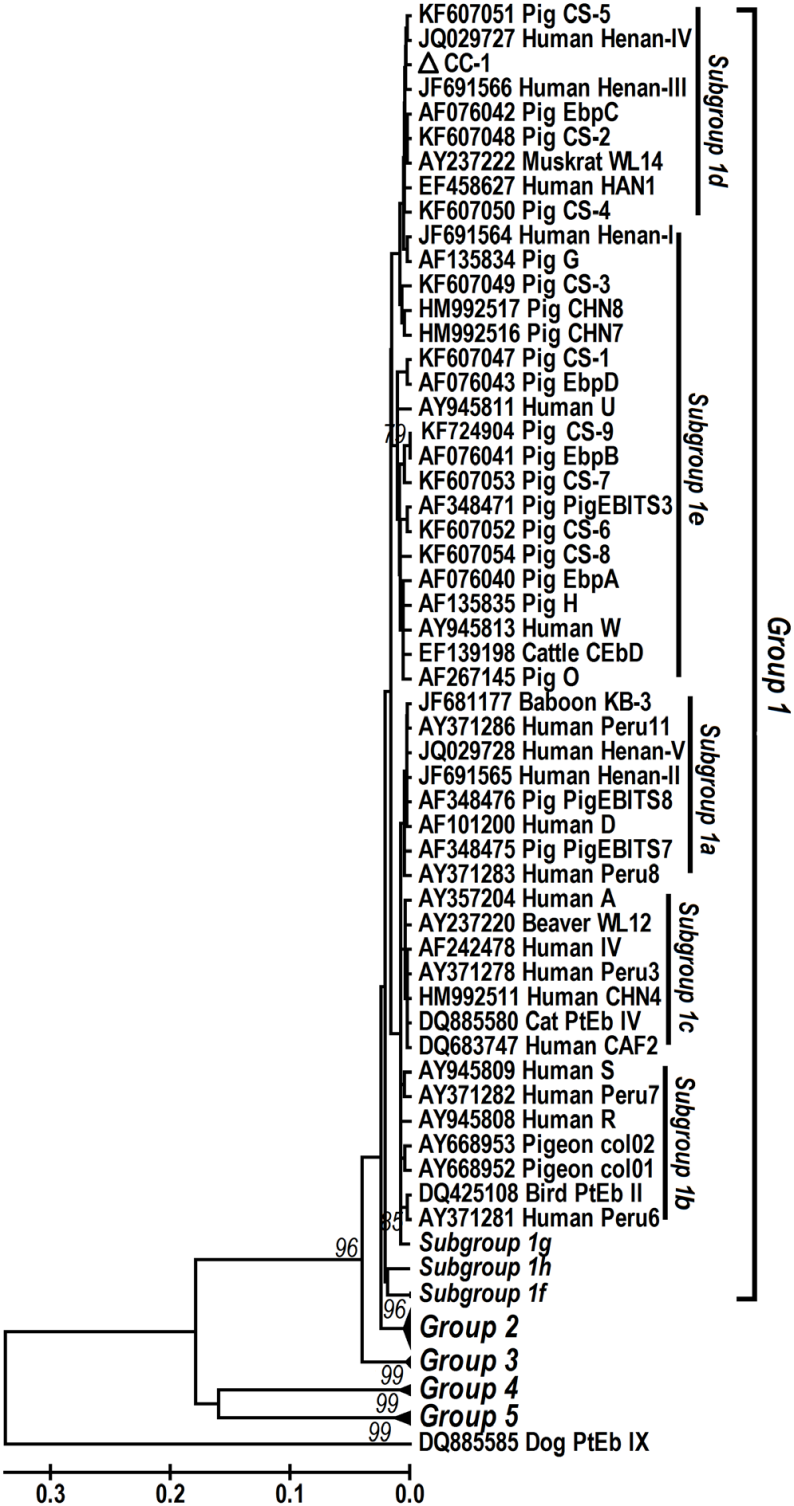
Phylogenetic relationship of ITS nucleotide sequences of *Enterocytozoon bieneusi* in this study and known *E. bieneusi* genotypes, as inferred by a neighbor-joining analysis (Mega 4 software [http://www.megasoftware.net/]) based on genetic distances calculated using the Kimura two-parameter model. The ITS tree was rooted with GenBank sequence DQ885585. Bootstrap values less than 65% from 1,000 pseudoreplicates are not shown. CC-1 indicated by white triangle is a new genotype found in this study.

Phylogenetic assessment of *E. bieneusi* genotypes from various sources revealed the clear clustering of the novel genotype CC-1 into one major genetic group (Group 1) reported by [7] (Fig. 2). The major cluster harbored the genotype CC-1 identified herein, genotype D in falcons in Abu Dhabi [12], genotypes Peru6 and PtEb II in pet birds and pigeons in Portugal [9], genotype EbpA in pet birds and pigeon in Brazil [10], genotypes A and EbpA in pet birds [11], col01 and col02 in pigeons [13] in Spain, genotypes CHN7, CHN8, D, EbpA, EbpC, EbpD, H, Henan-I, Henan-III, Henan-IV, O, and CS-1 to CS-8 in pigs in northeast China [25], [27], genotypes D, EbpC, EbpD, IV, Peru 8, Peru 11, PigEBITS7, and Henan-I to Henan-V in humans in Henan [28], genotypes EbpA and EbpC in humans in Shanghai [29], genotype CHN4 in humans in Changchun [27], and some other genotypes previously reported in humans and wild and domestic animals [1] (Fig. 2).

## Discussion

Although infections of *E. bieneusi* were frequently documented in humans, livestock, and wildlife around the world, very few studies have described the identification of this organism in birds (fowl birds, pet birds, pigeons, and falcons) [1], [8]–[13], [31]. This study examined *E. bieneusi* in baby chickens in 2 cities in northeast China in spite of a previous report describing its presence in chickens in Germany [8]. Our one previous study has revealed the presence of zoonotic *E. bieneusi* genotypes with high incidence and genetic diversity in diarrheic pigs in northeast China [25]. The sampling date in that study [25] ranged from September 2012 to April 2013, the present study extended the study date to another 3 months to make the study regarding the molecular surveillance of the neglected pathogen in diarrheic swine in northeast China more consummate. This study confirmed the infections of *E. bieneusi* in diarrheic pigs in city Tianjin and 6 other cities in northeast China. It is of interest to notice the difference of infection rates between this (17.2%; 11/64) and our former study (45.1%; 51/113) [25], which may attribute to the variances in seasonality and ecological environments.

The evaluation of zoonotic transmission of microsporidiosis between humans and animals chiefly depends on the improvement of genotypic identification of *E. bieneusi* from various host species and geographical regions by sequence analysis of the ITS locus [1], [3]. This study indicated the existence of 4 genotypes in chickens (CC-1 and Henan-IV) and pigs (CHN7, EbpC, and Henan-IV) in northeast China, with EbpC and Henan-IV previously found in human infections in Henan province [28], EbpC in hospitalized children in city Shanghai [29], EbpC and Henan-IV in pigs in several cities in northeast China [25], CHN7 in pigs in city Changchun [27], EbpC in wastewater in cities Wuhan and Qingdao in central China [4]. There were also some reports describing the infections of genotype EbpC in humans in Vietnam, Thailand, Peru, and Czech Republic [32]–[37]. In addition to the human-pathogenic genotypes EbpC and Henan-IV we identified, genotype CHN7 and the new genotype CC-1 are members of Group 1, thus have zoonotic potential.

Most of the *E. bieneusi* genotypes identified in birds have been previously reported in cases of human infections. For instance, genotype J previously reported in chickens in Germany infected humans in northeast China [8], [27]. Genotype D identified in falcons in Abu Dhabi was seen in humans in central China and many other countries [1], [12], [28]. Genotype Peru6 in pigeons and one pet bird in Portugal was detected in AIDS patients in Peru [9]. Genotype EbpA in pet birds and pigeons in Brazil and pet birds in Czech Republic also existed in humans in Nigeria and Czech Republic [38], [39]. Genotype A in pet birds in Czech Republic was previously reported in humans in many countries [1]. Therefore, birds could be a significant source of environmental contamination and potential source for human infection. In this study, we identified the occurrence in chickens a genotype Henan-IV previously found in an AIDS patient in Henan [28] and a new genotype CC-1 that was genetically clustered into Group 1. To the best of our knowledge, this is the first report that diarrheic chickens were infected with zoonotic *E. bieneusi* genotypes.

In conclusion, we reported the occurrence of *E. bieneusi* in diarrheic chickens in 2 cities and pigs in 7 cities in China. Identification of the human-pathogenic *E. bieneusi* genotypes Henan-IV (chickens and pigs) and EbpC (pigs) and the zoonotic genotypes CHN7 (a pig) and CC-1 (a chicken) suggests that chickens and pigs could be reservoirs for human microsporidiosis. Actions should be made to reduce the opportunities for close contact between *E. bieneusi*-harboring chickens and pigs and susceptible human populations in order to limit the spread of microsporidiosis.
